# Brain and behavioral correlates of action semantic deficits in autism

**DOI:** 10.3389/fnhum.2013.00725

**Published:** 2013-11-08

**Authors:** Rachel L. Moseley, Bettina Mohr, Michael V. Lombardo, Simon Baron-Cohen, Olaf Hauk, Friedemann Pulvermüller

**Affiliations:** ^1^MRC Cognition and Brain Sciences UnitCambridge, UK; ^2^Department of Psychiatry, Charite UniversitätsmedizinBerlin, Germany; ^3^Autism Research Centre, Department of Psychiatry, University of CambridgeCambridge, UK; ^4^Brain Language Laboratory, Department of Philosophy and Humanities, Freie Universität BerlinBerlin, Germany

**Keywords:** Autism, semantics, motor systems, action

## Abstract

Action-perception circuits containing neurons in the motor system have been proposed as the building blocks of higher cognition; accordingly, motor dysfunction should entail cognitive deficits. Autism spectrum conditions (ASC) are marked by motor impairments but the implications of such motor dysfunction for higher cognition remain unclear. We here used word reading and semantic judgment tasks to investigate action-related motor cognition and its corresponding fMRI brain activation in high-functioning adults with ASC. These participants exhibited hypoactivity of motor cortex in language processing relative to typically developing controls. Crucially, we also found a deficit in semantic processing of action-related words, which, intriguingly, significantly correlated with this underactivation of motor cortex to these items. Furthermore, the word-induced hypoactivity in the motor system also predicted the severity of ASC as expressed by the number of autistic symptoms measured by the Autism-Spectrum Quotient (Baron-Cohen etal., 2001). These significant correlations between word-induced activation of the motor system and a newly discovered semantic deficit in a condition known to be characterized by motor impairments, along with the correlation of such activation with general autistic traits, confirm critical predictions of causal theories linking cognitive and semantic deficits in ASC, in part, to dysfunctional action-perception circuits and resultant reduction of motor system activation.

## INTRODUCTION

A surprising finding in contemporary neuroscience concerns the motor system’s function as a vehicle for higher cognitive processes which, on first glance, appear to be entirely unrelated to basic motor function. Theoretical bridges between supposedly “lower-order” sensorimotor functions and cognitive processes, such as developing semantic concepts or language, have been proposed in the psychological literature (Piaget, 1950). Recent research indicates that this link may lie in action-perception circuits, neuronal ensembles connecting neurons in sensory and motor areas via brain systems intertwining the two (Pulvermüller, 1999; Fuster, 2003; Rizzolatti and Craighero, 2004; Jeannerod, 2006; Pulvermüller and Fadiga, 2010). Correlated activation in motor and sensory areas of the cortex is proposed to lead to the development of neuronal assemblies that represent motor acts. These action-perception circuits become the basis of mirroring, i.e., repeating visually perceived actions performed by others, repeating verbal utterances and working memory (Fuster, 2003; Rizzolatti and Craighero, 2004; Jeannerod, 2006).

**FIGURE 1:**
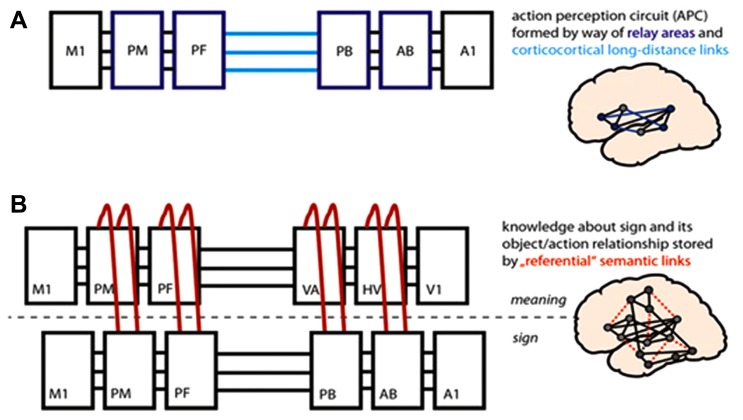
**Brain-model of action perception circuits (APCs) and semantic mechanisms.** **(A)** Based on corticocortical connections and correlated activity patterns in primary motor and auditory areas (M1, A1), APCs develop for spoken word forms; these include neurons in primary areas and in “relay areas” bridging between them [inferior PM (premotor) and PF (prefrontal); superior-temporal AB (auditory belt) and PB (parabelt)]. **(B)** Object-related and action-related semantic information is bound to word forms by way of long-distance links between APCs for word forms and concepts; the illustrated conceptual circuit involves neurons in dorsolateral M1, PM, and PF motor/executive cortex and in V1 (primary visual), HV (higher visual), and VA (visual association) cortex in the ventral object processing stream. The APC model predicts that lesions in motor areas (M1, PM) or disconnection of these regions leads to disintegration of the mechanisms for language processing and especially impacts on action-related semantics.

Critically, however, by interlinking with each other, such action-perception circuits can themselves become substrates for a range of additional higher cognitive processes, such as language and representation of conceptual meaning. Words denoting concrete, visible concepts, such as actions or visible objects, tend to be learnt in the context of interacting with or experiencing that concept in the world (Pulvermüller, 1999). In accordance with Hebbian principles, simultaneous activation across numerous brain regions results in the formation of connected circuits. Specifically, the sensorimotor patterns for hearing and articulating a word (represented in core perisylvian language areas, **Figure 1A**) become linked to the differential areas activated by experiencing/interacting with actions or objects, thus forming conceptual circuits for words. Action words such as “grasp,” which semantically relate to the concepts of actions represented by action schemas stored in cortical motor systems, therefore draw upon motor systems, whilst object words relate to visual objects and are thus processed in the temporo-occipital visual processing stream. This is depicted in **Figure 1B**, (but please see Garaghani et al., 2009 for elaboration on this model and the linkage of regions through Hebbian processes). Though this relates to concrete items, there may be a critical role for action-perception circuits in the representation of meaning for abstract words, too (Moseley et al., 2012). In addition, elementary action schemas can be linked into action chains and may multiply into action hierarchies, thus embedding actions and object representations into plans and frames (Pulvermüller and Fadiga, 2010). Research in social neuroscience has also repeatedly shown that action-perception systems involved in mirroring can interact synergistically with mentalizing systems (Zaki et al., 2009; Lombardo et al., 2010a; Schippers et al., 2010; Spunt and Lieberman, 2012). This mutual link suggests that motor problems may lead developmentally to a whole host of downstream deficits in higher cognitive functions (e.g., language, communication, social cognition, understanding action concepts, and the meaning of action words; Pulvermüller, 2005; see **Figure 1**).

If action-perception circuits form one basis of higher cognition, a motor deficit could hinder their formation and thus compromise, or alter, higher processes normally built upon the latter. Critical test cases here are patients with motor impairments, such as in stroke, Parkinson’s disease, or Motor Neuron disease, who indeed show specific deficits in understanding action-related concepts and in processing action-related words (Neininger and Pulvermüller, 2003; Boulenger et al., 2008; Bak and Chandran, 2011; Kemmerer et al., 2012). On this basis, it is however difficult to confidently attribute the motor systems a critical role in cognition: lesions are typically characterized by substantial involvement of multiple regions which limits linkage of cognitive deficits to this focal area. A syndrome marked by more subtle motor deficits and a broad range of social and cognitive difficulties may allow testing of new predictions regarding the role of sensorimotor systems in higher cognitive processing and corroborate the evidence provided by other neuropsychological patient groups, especially if behavioral experiments are combined with spatially precise neuroimaging.

A case in kind are autism spectrum conditions (ASC), neurodevelopmental conditions primarily diagnosed by the “triad” of social-communication deficits, stereotyped/repetitive behaviors, and unusually restricted/narrow interests. Interestingly, ASC are also commonly characterized by subtle motor impairments in gait, posture, fine and sometimes gross coordination (Jansiewicz et al., 2006; Dewey et al., 2007; Dziuk et al., 2007)*. *Perhaps due to the somewhat hidden nature of any potential link between social-communicative deficits and non-social motor abnormalities, the latter have traditionally been seen as minor, secondary phenomena, especially as the social-communicative deficits of autism are far more disabling. Emerging evidence, however, supports the idea that sensorimotor abnormalities *precede* the emergence of core social-communicative problems in infants at risk for developing autism (Teitelbaum et al., 1998; Rogers, 2009) and that later deficits in social interaction, imitation, and social cognition may emerge downstream from the atypical development of sensorimotor systems (Mostofsky et al., 2006). A further hint that socio-cognitive deficits and motor processes are linked comes from the mirror neuron literature, where inferior frontal and premotor “mirror neuron systems” (MNS) are hypoactive in ASC [Iacoboni and Dapretto, 2006; Cattaneo et al., 2007; Williams, 2008; Rizzolatti and Fabbri-Destro, 2010; though the role of the mirror system in autism is debated by other authors (Marsh and Hamilton, 2011)]. If a motor deficit precedes or is intertwined with higher cognitive deficits in ASC (Leary and Hill, 1996), the investigation of motor brain mechanisms, conceptual action understanding and severity of ASC may be fruitful in understanding the mechanisms of these complex conditions.

Dysfunction of action-perception circuits in ASC implies that these individuals should fail to activate action representations in speech comprehension, especially during understanding of action-related meanings (**Figure 1**). In contrast, when typically developing (TD) participants read action words or sentences, motor activation reflects somatotopic aspects of word meaning (Hauk et al., 2004; Pulvermüller and Fadiga, 2010). If action-perception circuits are indeed a basis for understanding action-related language, a further prediction is that people with ASC should exhibit a specific deficit in semantically understanding action words. Crucially, behavioral and motor-cognitive brain activation deficits should correlate with and should predict autistic traits.

To test these predictions of the action-perception model, we used event-related fMRI to assess patterns of cortical activation during passive reading and comprehension of action and object words in individuals with ASC and TD controls, and carried out a behavioral experiment to assess their ability to semantically classify these words. To elucidate links across different levels of brain and behavior, we then investigated correlations between motor system activation and cognitive-semantic ability, and also the correlation between activation and the number of autistic traits as measured by the Autism Spectrum Quotient (AQ; Baron-Cohen et al., 2001). Employing a data-driven regions of interest (ROI) approach, we were able to investigate activation evoked by action- and object-related words in frontal and temporal areas typically involved in language (Pulvermüller, 1999; Cohen et al., 2002; Kronbichler et al., 2004) and regions in the motor system which typically respond to action words (Pulvermüller and Fadiga, 2010). Given the movement impairments and structural abnormalities of motor systems in ASC, we hypothesised that these individuals would show a category-specific abnormality during the processing of words with action meaning. If motor systems play an important role in retrieving the meaning of action words, a processing deficit for these words manifested in psycholinguistic semantic tasks should be predicted by abnormal activity for action words in this motor region.

## MATERIALS AND METHODS

### PARTICIPANTS

Nineteen participants with ASC were initially recruited for the study. One fMRI dataset was lost due to excessive movement, and so brain activity of 18 participants with ASC [mean age: 30.4 SD: 10, range: 39; mean IQ: 113.5 (SD: 23)] was compared with that of 18 typically-developed controls [mean age: 28.6 (SD: 11.7, range: 44); mean IQ: 110.2 (SD: 12.3)], with all participants being right-handed monolingual native speakers. No significant differences appeared between the groups in age [*t*(34) = 0.490, *p* > 0.6)] or IQ [*t*(34) = 0.411, *p* > 0.6), and they were roughly balanced for gender (9 men in the ASC group, 12 men in the control group). All ASC participants (17 with Asperger Syndrome, 1 with PDD-NOS) were recruited from the participant panel of the Autism Research Centre (ARC) in Cambridge, where they were registered after having been clinically diagnosed using DSM-IV criteria. The ASC group scored significantly higher than the control group [*t*(32) = 6.857, *p* < 0.001] on the Autism Spectrum Quotient (AQ: Baron-Cohen et al., 2001), with a mean score of 34 (SD: 10) in comparison to 13 (SD: 5). All but 4 of the ASC group scored above 26 on this test, a cut-off point believed to capture the majority of adults with autism (Woodbury-Smith et al., 2005). The same authors found the AQ to reliably discriminate between individuals with and without autism (correctly classifying 83% of individuals). The study was approved by the NRES Cambridgeshire 3 Ethics Committee.

### STIMULI

Critical stimuli employed in the study included 120 action-related (e.g., “grasp,” “walk,” “chew”) and 120 object-related (e.g., “cheese,” “shark,” “flute”) words without inflections. Prior to the fMRI experiment, a semantic rating study was carried out (please see Hauk et al., 2004 for full details of procedure) on a large corpus of words to ascertain semantic features including imageability, concreteness, visual-relatedness, form-relatedness, color-relatedness, arousal, valence, and action-relatedness. Words were matched for psycholinguistic factors including word frequency, letter bigram and trigram frequency, number of orthographic neighbors, and number of meanings. Please see **Table 1** for psycholinguistic and semantic features of critical stimuli. In order to distract participants from the study’s focus on action- and object-language, they were interspersed with 120 filler words (e.g., “fluke,” “ail,” “cite,” which were matched to experimental words in length, bigram and trigram frequency, and number of neighbors) and 120 hash-mark strings (###), which, also matched for length, acted as a low-level visual baseline.

**Table 1:** Psycholinguistic and semantic features of word stimuli.

	Action words	Object words	Main effect of word type (*t*)	Filler words
Length	4.50 (0.066)	4.34 (0.063)	1.660 (*p* > 0.09)	4.58 (0.061)
Word frequency	11.38 (1.43)	10.22 (1.32)	0.597 (*p* > 0.55)	10.35 (1.76)
Bigram frequency	33083.89 (1644.70)	37313.48 (1532.15)	-1.882 (*p* > 0.06)	39029.90 (1669.87)
Trigram frequency	3465.95 (347.69)	4144.69 (342.33)	-1.391 (*p* > 0.16)	4096.74 (436.24)
No. of neighbors	7.13 (0.476)	7.88 (0.503)	-1.082 (*p* > 0.28)	6.41 (0.483)
No. of meanings	1.18 (0.040)	1.328 (0.067)	-1.845 (*p* > 0.06)	1.05 (0.023)
Imageability	4.44 (0.084)	5.82 (0.098)	-10.701 (*p* < 0.001)	2.59 (0.116)
Concreteness	3.69 (0.068)	6.24 (0.065)	-27.155 (*p* < 0.001)	2.95 (0.084)
Visual-relatedness	3.86 (0.108)	5.88(0.073)	-15.457 (*p* < 0.001)	2.13 (0.112)
Form-relatedness	2.46 (0.092)	3.25 (0.074)	-6.722 (*p* < 0.001)	1.40 (0.059)
Color-relatedness	1.56 (0.059)	2.30 (0.112)	-5.793 (*p* < 0.001)	1.21 (0.053)
Arousal	3.13 (0.095)	1.41 (0.056)	15.625 (*p* < 0.001)	2.02 (0.094)
Valence	3.83 (0.092)	3.86 (0.043)	-0.245 (*p* > 0.87)	3.22 (0.117)
Action-relatedness	5.31 (0.085)	2.10 (0.118)	22.096 (*p* < 0.001)	3.91 (0.151)

*Statistical tests between action and object words are displayed in t values. The psycholinguistic properties of the filler words are included in the rightmost column but not in the statistical tests reported, as they were included to detract from the nature of the task and were not compared with the experimental word categories.*

The full-display of the monitor presenting the stimuli had a visual angle of 16.7° (width display 25.16°, height display 14.31°). The stimuli were presented subtending a visual angle of 2.3°.

### PROCEDURE

For this task of silent reading, subjects were scanned in a 3-T Tim-Trio scanner with a 12-channel head-coil attached. Functional scans consisted of 32 slices covering the whole brain in descending order (slice thickness: 3 mm, in-plane resolution: 3 mm × 3 mm, inter-slice gaps: 0.75 mm), and echo-planar sequence parameters were TR = 2000 ms, TE = 30 ms, and flip angle = 78°. The silent reading task was split into three EPI blocks of approximately 7 min and 210 32-slice volumes each, with five dummy scans used at the beginning of each block to achieve a T1-steady state but discarded in the analysis.

Brain activity was compared between groups during passive reading of action- and object-related words. These stimuli, interspersed with filler words and hash-mark strings, were projected onto a screen and presented for 150 ms in a randomized order, with a 2.5-s stimulus onset asynchrony, and participants were requested to keep as still as possible, attend to the stimuli, and read them silently. This task was split into three parts of approximately 7 min each (21 min overall), allowing participants breaks in between if needed. Following the scan and without prior warning, they performed a word recognition test (involving rating a list to indicate their recognition of novel words and some of those previously seen in the experiment) that confirmed that they had been attentive during scanning. The data confirmed that they had been attentive: both groups performed above chance [average hit rate: controls = 76.2% (SD = 18.1%), ASC = 76.2% (SD: 19.1%)], with no significant difference appearing between them in the number of correct answers [*t*(34) = -0.018, *p* > 0.9].

Participants returned 4–10 weeks later (average: 8 weeks) to perform a semantic decision experiment on the action- and object-related words previously used in the fMRI experiment. Their task was to indicate as quickly as possible, within an interval of 2.5 ms, whether the meaning of tachistoscopically presented words (150 ms) related to actions or objects by button presses with the left or right thumb (counterbalanced over participants). Words were presented in light gray font on a black monitor; the order was pseudo-randomized between participants. After completing the semantic decision task, participants completed the Autism Spectrum Quotient (Baron-Cohen et al., 2001).

To compensate for drop-outs, two new ASC and seven TD controls were recruited. Altogether, 19 ASC and 18 TD subjects took part in the behavioral experiment, as the one ASC individual whose fMRI dataset was excluded was included in this analysis. Age and IQ differences between groups remained non-significant.

### DATA ANALYSIS

SPM5 (Wellcome Department of Imaging Neuroscience, London, UK) was employed for all processing stages, including slice-timing and re-aligning using sinc interpolation, co-registration of images to structural T1 images and normalization of the previous to the 152 subject T1 template of the Montreal Neurological Institute (MNI). Transformation parameters were applied to co-registered EPI images, which were also resampled with a spatial resolution of 2 mm × 2 mm × 2 mm and spatially smoothed with an 8-mm full-width half-maximum Gaussian kernel.

Single-subject statistical contrasts were computed using the canonical hemodynamic response function (HRF) of the general linear model. Low-frequency noise was removed by applying a high-pass filter of 128 s. Onset times for each stimulus were extracted from Eprime output files and integrated into a model for each block in which each stimulus category was modeled as a separate event. Group data were then analyzed with a random-effects analysis and second level group analysis performed. Activation to each of the experimental word categories in each groups was compared statistically against baseline (the hashmark condition) and voxel coordinates reported in MNI standard space.

In addition to whole-brain analysis, a ROI investigation was undertaken using the MarsBar function of SPM5. As the left hemisphere is the major site of language processing, four 2 mm-radius regions located in left-hemispheric key areas of theoretical interest from previous literature (inferior frontal gyrus, superior temporal sulcus, precentral and fusiform gyrus) were extracted from the contrast of all words against baseline (###) in typical controls, and four right-hemispheric homologs were chosen to match these as closely as possible. Three of the four ROIs were also confirmed by the activation patterns seen when both groups were pooled (a highly significant peak in the superior temporal sulcus had marginally different coordinates). Note the fact that the all-words vs. baseline contrast which is orthogonal to the contrasts relevant for hypothesis testing (ASC vs. TD) rules out the risk of double dipping (see also Kriegeskorte et al., 2009). Activation for action- and object-word categories was compared between groups in these regions. Because voxels were resampled with a spatial resolution of 2 mm × 2 mm × 2 mm and smoothed at a 8 mm kernel, the half maximum width of each 2 mm-radius ROI was 12 mm, thus allowing us to keep ROIs overlap free while at the same time compensating for some of the spatial variance caused by the projection of individual brains to the averaged MNI template. Statistical analysis of ROIs was executed in both SPSS and Statistica. Bonferroni corrections were applied on the data where appropriate and are indicated in the text. For the main 4-way ANOVA, correction was for the full 15 significance tests.

## RESULTS

### fMRI RESULTS: FRONTAL-MOTOR HYPOACTIVITY IN ASC

**FIGURE 2:**
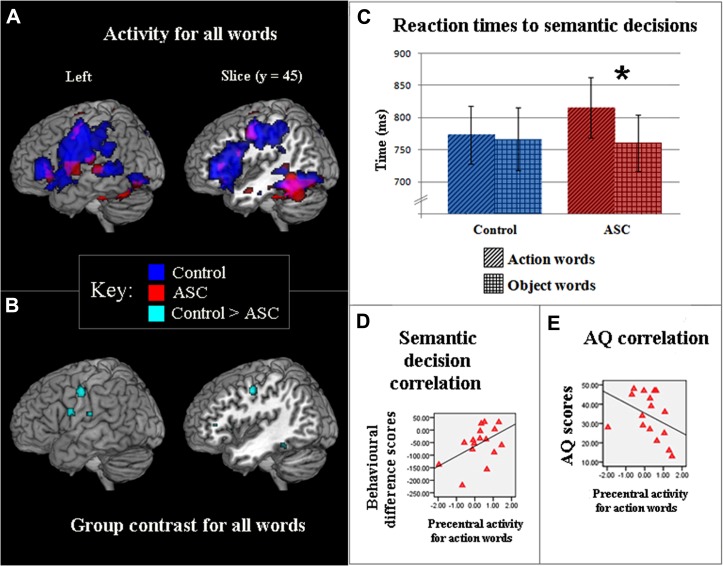
**(A)** Activity for words (*p* < 0. 005, uncorr.) viewed in a passive reading task contrasted against a hash mark (###) baseline, in TD controls (blue) and participants with ASC (red). **(B)** Direct statistical contrast between groups for words viewed in the passive reading task (FWE *p* < 0.05). Light blue foci show areas of stronger activation for TD controls as compared to ASC (TD > ASC) participants: the opposite contrast, ASC > TD, was non-significant. **(C)** Latencies (ms) of TD controls (blue) and ASC participants (red) who made semantic classifications for action- (diagonal stripes) and object-related (crosshatch) words. Bars show average response times (and standard errors) taken to make semantic decisions. The significant difference between word categories in ASC is reflected by an asterisk (^*^). **(D)** Significant correlations for ASC participants between activity in the motor system for action words and behavioral difference scores in the semantic decision task. Behavioral underperformance in classifying action words was quantified by subtracting response times for matched object-words from those to action-related words, and is significantly correlated with lower activity in the motor system. Motor activation was measured in precentral cortex, at coordinate (-50, -10, 44), where maximal activation was seen in action word reading in TD participants. **(E)** Significant correlations for ASC participants between activity in the motor system [see **(D)**] for action words and AQ scores. Higher numbers reflect increasing number of autistic traits. In **(C,D)**, values along the *x*-axis reflect parameter estimates (arbitrary units) reflecting the difference in activation between action words and the baseline condition (hash-marks).

In both groups (18 ASC vs. 18 TD participants), the contrast of all words against baseline [strings of repeated familiar symbols (hash marks)] revealed similar activation patterns in posterior temporal regions, which are typically activated by written word stimuli (Cohen et al., 2002). In contrast, inferior-frontal and precentral cortex were strongly active in TD controls but not in people with ASC. This finding was revealed by a low level contrast (words vs. baseline) and is displayed at a lenient threshold (uncorrected *p* < 0.005) in **Figure 2A** to show the full activation range for both groups. Following stringent whole-brain correction for multiple comparisons (*p* < 0.05, FWE corrected), direct statistical comparison of word-elicited activations between both groups still confirmed that ASC subjects showed reduced inferior-frontal and precentral activation compared with TD controls (**Figure 2B**). The opposite contrast (ASC > TD) failed to reach significance anywhere in the brain.

To explore whether between-group differences were significantly more pronounced in frontal cortex compared with other sites, a ROI analysis and ANOVA were conducted on data from the two frontal and two temporal regions which emerged from the contrast of all words against baseline (###). These were included in a four-way ANOVA, including the two-level factors “hemisphere,” “peris- vs. extrasylvian” (“PES”), “frontal vs. temporal” (“FT”) and the group variable. A significant interaction of factors PES, FT, and Group [*F*(1, 34) = 9.234, *p* < 0.01] revealed significant differences in word-related activity between groups, with generally lower activity for autistic subjects but particularly strongly reduced activity in frontal cortex. Further exploration of the language-dominant left hemisphere revealed a significant interaction of the factors Fronto-temporal and Group [*F*(1, 34) = 4.210, *p* < 0.05], which further confirmed specificity of hypoactivity to inferior-frontal and precentral sites in the ASC group. Following Bonferroni-correction, significant between-group differences were only found in the deep-inferior frontal [*t*(34) = 4.229, *p* < 0.001] and precentral gyrus [*t*(34) = 3.514, *p* < 0.002], but not in temporal areas.

**FIGURE 3:**
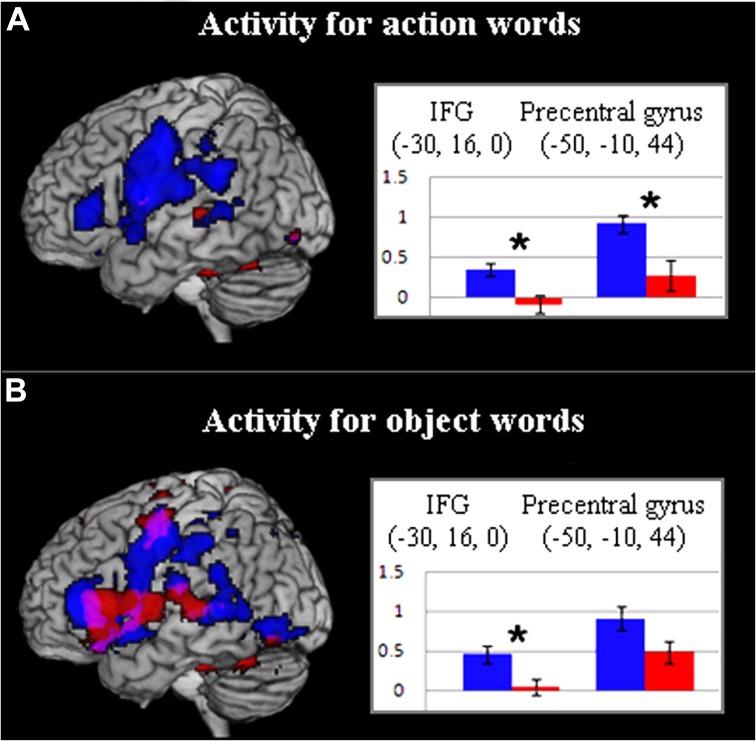
**(A)** Activity for action words in control (blue) and ASC (red) participants (*p* < 0. 005, uncorr.). Bar charts depict action-word activity for both groups in key inferior frontal and precentral ROIs. **(B)** Activity for object words in control (blue) and ASC (red) participants (*p* < 0.005, uncorr.). For further explanation, see legend for **(A)**. In **(A,B)**, values along the *y*-axis reflect parameter estimates (arbitrary units) reflecting the difference in activation between action or object words respectively and the baseline condition (hash-marks).

Since motor systems are activated during passive speech perception and language comprehension (Wilson et al., 2004; D’Ausilio et al., 2009; Pulvermüller and Fadiga, 2010), this group difference in word-elicited motor activation could reflect an ASC-specific processing difficulty in mapping language to articulatory motor programes. However, separation of hemodynamic response by word type in the previously defined ROIs confirmed ASC-specific frontal hypoactivity for action words but only partially for object-related words. Although inferior-frontal cortex showed between-group differences for both word types, precentral cortex revealed a significant group difference, with reduced activity in the ASC group, for the contrast of action words against baseline [*t*(34) = 2.917, *p* < 0.01], but not for object-related words against baseline (**Figures 3A,B**). Whilst the interaction of group and word category was non-significant (*p* < 0.1), these results do suggest that reduced motor system activation in ASC relates to semantic-conceptual processing. Though this finding is consistent with the study hypotheses, the lack of a group difference for object words in the motor system could potentially reflect a failure of statistical power and this might be further investigated in future experiments. At present, however, only behavioral data can clarify whether such activation is *necessary* for semantic processing: if precentral/premotor hypoactivity in ASC reflects a genuine semantic processing deficit in motor cognition, this should be apparent during processing of action-related words.

### BEHAVIORAL RESULTS: LINKING BRAIN WITH BEHAVIOR

To address this question experimentally, participants from the fMRI experiment returned to perform a semantic decision experiment on the same action- and object-related words. Due to drop-outs and the loss of some datasets, this analysis also included three ASC participants who were not included in the fMRI study (see Procedures, above, for details). A factorial two-way ANOVA (Word category × Group) showed that performance was generally high throughout the task, without revealing a difference between groups or word kinds [TD: mean = 87% correct, SD = 5%; ASC: mean = 87% correct, SD = 5.5%; *F*(1, 35) = 0.128, *p* > 0.7]. However, an ANOVA performed on reaction times revealed a significant interaction between Word category and Group [*F*(1, 35) = 4.291, *p* < 0.05]. ASC participants were significantly slower in semantically-judging action-related words compared with their speed at semantically classifying object words [*t*(18) = 3.116, *p* < 0.01]; TD individuals showed no evidence of a similar contrast [*t*(17) = 0.429, *p* > 0.6], **Figure 2C**). These results show that, in a speeded semantic decision task, ASC participants are significantly debilitated in processing action-related words (mean: 815.3 ms, SD: 204.5) compared with matched object words (mean 760.6 ms, SD: 191.5).

The strongest *a priori* prediction action-perception theory makes about ASC concerns the relationship between semantic processing deficits and reduced motor system activation in cognitive processing, so correlations between behavioral response times and cortical activation in the left-premotor ROI (-50, -10, 44) during action word reading were examined in datasets from the 16 ASC participants who participated in both experiments. To obtain a specific behavioral measure of action semantics, we used the object word response times for normalizing the action word latencies in semantic decisions. A significant correlation between reaction time and precentral activation to action words (*r* = 0.497, *p* < 0.05) was observed in the ASC group, whereby relative underperformance on the semantic task for action verbs, but not object nouns, was linearly related to decreasing brain activation elicited by action words (**Figure 2D**). Further exploration of the behavioral-BOLD correlation in the previous ROIs revealed a similar correlation for inferior frontal cortex with action words, but not for other parts of the brain. No comparable correlations with brain activity were observed for object words.

In order to explore the link between activity in semantic motor system activity and the wider spectrum of autistic symptoms, we studied the correlation between precentral semantic activity and autistic symptoms as assessed by the AQ (Baron-Cohen et al., 2001). Higher scores on the AQ (greater number of autistic traits) were significantly correlated with hypoactivity in the same precentral ROI cortex for words generally (*r* = -0.556, *p* < 0.02). Following removal of one marked outlier with a family history of left-handedness (seen to the far left in **Figure 2E**), this negative correlation remained significant. The correlation was especially pronounced when considering brain activity elicited by action words alone (*r* = -0.654, *p* < 0.005; **Figure 2E**). All other analyses’ remained significant with removal of this individual and the ASD group were still significantly slower to process action words [*t*(17) = 2.797, *p* < 0.02].

## DISCUSSION

Here we report a novel investigation of semantic action word processing in ASC and its relationship to motor cortex activation and general ASC symptomatology. Using fMRI, we found hypoactivation of inferior-frontal and premotor cortex during word reading in ASC relative to matched control participants. This reduction in activity was most clearly apparent for words semantically related to actions. Corresponding to the significantly reduced activation of motor systems in action word processing seen in ASC, we found increased reaction times for processing these words in this group, thus indicating a category-specific abnormality in semantic processing. Third, linking the two results together, a significant correlation emerged between hypoactivity in the motor system and slowed reaction time for processing action words. Critically, a similar correlation also appeared between semantic hypoactivity and autistic symptomatology in our ASC group. These results all support the prediction of an action-perception theory of ASC, whereby the reported category-specific semantic processing disadvantage, and possibly a wider range of ASC symptoms, may stem from atypical information exchange between the motor cortex and other brain regions.

This newly observed language-related hypoactivation of motor systems in ASC and their correlated deficit in semantically processing action-related words refute an interpretation of semantic motor systems activation as “ancillary” or “epiphenomenal” in the general population. Rather, it appears that an intact and well-connected motor system brings about motor system activation during action word reading and is necessary for optimal processing of these items, whereas, in a condition where this activation is absent, specific abnormalities in semantic information processing are manifest for words with action-related meaning. “Disembodied” theories of conceptual representation (Mahon and Caramazza, 2008), assuming meaning processing divorced from sensorimotor systems, cannot account for this finding. Nor is there currently evidence to suggest the activation of motor systems by another region or system (see Kiefer and Pulvermüller, 2012, for review and discussion of the literature). Although a range of cortical areas take their share in meaning processing (Pulvermüller, 2013), the present study failed to reveal brain activation outside the motor system that predicted the ASC-specific deficit in semantic processing of action-related words.

To account for the observed correlations between motor system hypoactivity, action-semantic deficit and ASC symptomatology, parsimony demands that a causal link be postulated. That socio-communicative or semantic deficits in autism might give rise to motor impairments seems unlikely, given that such a proposition would fail to explain why semantic deficits are specific to action words or why premotor cortex is hypoactive in language processing; furthermore, this position seems difficult to reconcile with the early emergence of motor dysfunction in ASC, long before semantic or social deficits become manifest or at least evident to current means of measurement at this age (Teitelbaum et al., 1998; Rogers, 2009). As there is currently no evidence for a third process acting on both motor and semantic systems, the third possibility is offered by action-perception theory. A functional deficit in motor areas and/or in the interaction between motor and other brain systems (see **Figure 1**), leads to atypical development of action-perception circuits required for language processing, motor cognition and action semantics. Thus the ASC motor deficit, which emerges early in ontogenesis, would cause hypoactivity in the precentral cortex in action-semantic processing and the observed slowing of action-semantic classification in ASC. On the basis of the existing literature on ASC and the specificity of the present results, such an account is highly plausible.

The observed action-semantic deficits in autism are paralleled in patients suffering from lesions in the motor system (Neininger and Pulvermüller, 2003; Boulenger et al., 2008; Bak and Chandran, 2011). The significant advance of the present study is the specificity of the relationship between cognitive-semantic deficits and the functionality of focal precentral cortex activation. The functional importance of motor systems for higher cognition demonstrated by earlier cognitive and brain research (Buccino et al., 2005; Pulvermüller et al., 2005; Fischer and Zwaan, 2008; Shebani and Pulvermüller, 2013) fits with a potential causal role of autistic motor dysfunction, as suggested by the developmental *primacy* of motor symptoms relative to core autistic symptomatology (Teitelbaum et al., 1998; Rogers, 2009). Indeed, impairments in the motor system in ASC and its connectivity predict not only movement problems but difficulty establishing typical action-perception circuits and therefore representations of complex actions (Mostofsky and Ewen, 2011). In turn, such representational alteration may lead to higher-order deficits in action understanding (Blake et al., 2003; Williams, 2008), gesture and imitation (Williams et al., 2001; Dewey et al., 2007), as well as language and communication. Although presently unascertained, it appears plausible that the aberrant structural connectivity of cortico-cortical tracts and reduced functional connectivity in ASC (Courchesne and Pierce, 2005; Sundaram et al., 2008; Jones et al., 2010) contribute to their motor and cognitive impairments. Atypical connectivity between frontal action-systems and posterior perception-related neural systems in the arcuate fascicles, which connect anterior and posterior language regions (Keller et al., 2007; Fletcher et al., 2010; Lai et al., 2012), may be of special relevance here, considering the key role these pathways play in connecting action-perception circuits, thus merging information about actions and perceptions in linguistic and semantic neural systems (Pulvermüller and Fadiga, 2010). The atypical development of language circuits would explain why, during reading words of different semantic categories, participants with ASC exhibit atypical patterns of local cortical activity (Moseley et al., 2013). We were unable, in the present study, to ascertain the degree of cortical motor abnormality in our participants, and this, alongside perhaps the course-grained measure of semantic processing in our behavioral task, might explain why inaccuracy in action word processing was not revealed alongside longer reaction times. The “tipping point” at which brain abnormalities manifest in semantic errors is likely to be influenced by task demands. It is clear that this study convincingly supports the contribution of motor regions to optimal action word processing, but much remains to be elucidated regarding the contribution of these and other brain areas to semantic processing in different contexts (Hauk and Tschentscher, 2013; Pulvermüller, 2013).

Action-perception circuits provide a functional link between sensory and motor neurons and become active when the individual performs an action and when they perceive the action visually or hear its characteristic action sounds (see **Figure 1B**). This mechanism explains the response patterns of mirror neurons, which play a key role in these circuits. Over and above providing a mechanism of mirror neuron activity and behavioral imitation, these action perception circuits can support a range of higher mental processes necessary for language, semantics, and social cognition (Pulvermüller and Fadiga, 2010). The lack of “embodied” action-related semantic processing in ASC demonstrated for the first time in this study is therefore in agreement with the well-known inactivity of the mirror neuron system seen in ASC subjects during tasks unrelated to language (Iacoboni and Dapretto, 2006; Cattaneo et al., 2007; Williams, 2008; Rizzolatti and Fabbri-Destro, 2010). In this context, the correlation between language-evoked motor activation and autistic traits (AQ scores) bolsters the previous suggestion that a range of typical ASC symptoms relate to motor systems abnormalities (Mostofsky et al., 2006; Mostofsky and Ewen, 2011). Although our present data are consistent with the prediction that an impairment of mirror mechanisms relying on action perception circuits would entail ASC deficits in semantic motor system activation, semantic processing of action words, and even general traits of ASC, we hasten to emphasize that that our data are correlational and therefore cannot provide proof of causality. Still, we offer some related considerations in the following paragraph.

Atypical grounding of semantics in action-perception circuits, such as would result in abnormal linguistic/communicative processing, might derail further development in domains that depend on input from motor systems, such as mentalizing. In particular, several researchers have hypothesized that “embodied” premotor cortical systems involved in mirroring also interact with systems for mentalizing (Zaki et al., 2009; Lombardo et al., 2010a; Schippers et al., 2010; Spunt and Lieberman, 2012). For example, reduced functional connectivity in ASC has been observed between a key mentalizing/self-representation region, ventromedial prefrontal cortex, and ventral premotor cortex and somatosensory cortex (Lombardo et al., 2010b). This would suggest that atypical development of (premotor-prefrontal links in) action-perception circuits underlying higher cognition could impact on the way in which individuals with ASC interact with others (Lombardo and Baron-Cohen, 2010,^2011^), and how such motor problems, preceding higher-level socio-communicative difficulties, might set children on atypical trajectories that lead to increased risk for autism. Though implications beyond the semantic processes studied in this present work may appear as speculative, our results clearly demonstrate that motor problems in ASC cannot be regarded as separate from, or secondary to, higher cognitive and socio-communicative difficulties. Instead, atypical development of action-perception circuits carrying higher cognitive processes derail aspects of language and conceptual processing which may entail further difficulties in communication, social interaction, and thought.

Further investigation is clearly necessary as to the relationship between motor system dysfunction and the development of other symptoms of ASC. One limitation of the present study is the lack of rigorous in-study diagnosis of ASC and of an overt behavioral measure of motor dysfunction. The inclusion criteria of our experiment strictly excluded those with “suspected” ASC and all individuals who had not received a previous formal diagnosis, and as such we were confident of the diagnostic status of our ASC participants. Here, the lack of motor systems response to word and action word processing was taken to reflect abnormality in these underlying systems, but future work might look in parallel at surface motor symptoms of such abnormalities, and relate them in greater detail to autistic symptoms as captured by gold-standard diagnostic instruments. Though movement abnormalities have been neglected in autism research, a causal dependence of cognitive and semantic capacities on motor systems, if demonstrated empirically, could have substantial implications for conceptualization of and interventions for ASC.

Whilst the majority of our discussion has focused on the deficit specific for action words in this population, a final note for consideration concerns the processing of visual object words. It has been suggested that individuals with ASC depend more on perceptual, perhaps more surface-level strategies of processing, rather than deep semantic analysis (Kamio and Toichi, 2000; Toichi and Kamio, 2001,^2002^,^2003^; Harris et al., 2006; Kana et al., 2006; Mottron et al., 2006; Gaffrey et al., 2007). Despite showing substantial activity in inferior temporal cortex, ASC participants did not activate this region significantly more than typically developed controls whilst reading, though the task in the present study is not directly comparable to previous findings as it involved passive reading and therefore minimal processing demands. Strength in visual or perceptual processing might, however, be supported by the relative sparing of visual object words in ASC participants, who were slower than controls at semantically judging action but not object words. In the typical population, object words also evoke activity in motor systems which relates in a somatotopic manner to the primary affordances of the concept denoted: tool words evoke activity in dorsal motor system (hand area) and the left cerebellar hemisphere which controls the right hand of the body, and food words activate dorsal portions of the motor system related to the face and mouth (Carota et al., 2012). Such motor activity, reflecting action semantic knowledge related to object affordances, appeared to be preserved here in ASC, a finding which sits parallel to the lack of a group difference in object word-induced brain activation. It appears that the direct linkage between an action word and the action it denotes is degraded in ASC, whilst the more indirect relationship between an object word and the affordances of the concept remains intact; but further investigation is required to assess this possibility and why, furthermore, object words might hold a privileged place in processing in ASC (at least compared with action words). The bias towards visual processing in ASC (Mottron et al., 2006) might provide a protective factor for words with primarily visual semantic associations. Another possibility is that action words, alongside their particular dependence on motor schemas, are additionally jeopardized by the social-pragmatic information intrinsic to their nature. Unlike object words, all action words imply an actor and several of the action words in this experiment had social associations (e.g., “speak,” “smile,” and “kiss”), and might therefore be specially problematic given the fundamental social handicap in ASC (Baron-Cohen, 2009). A third possibility is that the deficit seen here reflects a generic abnormality for processing the lexical verb class rather than an abnormality for words with action meaning *per se*. Strong neuropsychological and neurophysiological data suggests that the organization of meaning in the brain is driven by semantic rather than lexical differences (Vigliocco et al., 2011; Cappa and Pulvermüller, 2012; Kemmerer et al., 2012; Kiefer and Pulvermüller, 2012), but the present study cannot speak to this in ASC and so that, at present, we cannot refute with certainty the possibility that other morphosyntactic differences between nouns and verbs might result in the difference seen here between action and object words. Future research might choose to study different types of verbs (for example, abstract nouns and verbs such as “beauty” and “contemplate”) in ASC, to investigate whether the action word deficit observed in the present work relates to lexical category or to the action semantic content of these words.

## CONCLUSION

In contrast to TD control subjects, ASC participants do not significantly activate cortical motor-executive systems during language processing and show corresponding difficulties processing the action-related meaning of words. Crucially, motor hypoactivation predicted, and significantly correlated with, these semantic processing difficulties, consistent with a causal role of motor-executive systems in processing action-related meaning. Motor hypoactivity also predicted the severity of autistic traits, thus suggesting a further relationship between dysfunction of motor systems and wider traits typical in ASC. More research is needed to elucidate the putative role of neural motor systems in ASC and, more generally, in social cognition and theory of mind.

## Conflict of Interest Statement

The authors declare that the research was conducted in the absence of any commercial or financial relationships that could be construed as a potential conflict of interest.

## References

[B1] Bak T. H. Chandran S. (2011). What wires together dies together: verbs, actions and neurodegeneration in motor neuron disease. *Cortex* 48 936–944 10.1016/j.cortex.2011.07.008 21924711

[B2] Baron-Cohen S. (2009). Autism: the empathizing–systemizing (E–S) theory. *Ann. N. Y. Acad. Sci.* 1156 68–80 10.1111/j.1749-6632.2009.04467.x 19338503

[B3] Baron-Cohen S. Wheelwright S. Skinner R. Martin J. Clubley E. (2001). The autism-spectrum quotient (AQ): evidence from Asperger Syndrome/high-functioning autism, males and females, scientists and mathematicians. *J. Autism Dev. Disord.* 31 5–17 10.1023/A:1005653411471 11439754

[B4] Blake R. Turner L. M. Smoski M. J. Pozdol S. L. Stone W. L. (2003). Visual recognition of biological motion is impaired in children with autism. *Psychol. Sci.* 14 151–157 10.1111/1467-9280.01434 12661677

[B5] Boulenger V. Mechtouff L. Thobois S. Broussolle E. Jeannerod M. Nazir T. A. (2008). Word processing in Parkinson’s disease is impaired for action verbs but not for concrete nouns. *Neuropsychologia* 46 743–756 10.1016/j.neuropsychologia.2007.10.007 18037143

[B6] Buccino G. Riggio L. Melli G. Binkofski F. Gallese V. Rizzolatti G. (2005). Listening to action-related sentences modulates the activity of the motor system: a combined TMS and behavioural study. *Brain Res. Cogn. Brain Res.* 24 355–363 10.1016/j.cogbrainres.2005.02.020 16099349

[B7] Cappa S. F Pulvermüller F. (2012). Language and the motor system. *Cortex* 8 785–787 10.1016/j.cortex.2012.04.010 22579224

[B8] Carota F. Moseley R Pulvermüller F. (2012). Body-part-specific representations of semantic noun categories. *J. Cogn. Neurosci.* 24 1492–1509 10.1162/jocn_a_00219 22390464

[B9] Cattaneo L. Fabbri-Destro M. Boria S. Pieraccini C. Monti A. Cossu G. (2007). Impairment of action chains in autism and its possible role in intention understanding. *Proc. Natl. Acad. Sci. U.S.A.* 104 17825–17830 10.1073/pnas.0706273104 17965234 PMC2077067

[B10] Cohen L. Lehericy S. Chochon F. Lemer C. Rivaud S. Dehaene S. (2002). Language-specific tuning of visual cortex? Functional properties of the Visual Word Form Area. *Brain* 125 1054–1069 10.1093/brain/awf094 11960895

[B11] Courchesne E. Pierce K. (2005). Why the frontal lobe in autism might be talking only to itself: local over-connectivity but long-distance disconnection. *Curr. Opin. Neurobiol.* 15 225–230 10.1016/j.conb.2005.03.001 15831407

[B12] D’Ausilio A. Pulvermüller F. Salmas P. Bufalari I. Begliomini C. Fadiga L. (2009). The motor somatotopy of speech perception. *Curr. Biol.* 19 381–385 10.1016/j.cub.2009.01.017 19217297

[B13] Dewey D. Cantell M. Crawford S. G. (2007). Motor and gestural performance in children with autism spectrum disorders, developmental coordination disorder, and/or attention deficit hyperactivity disorder. *J. Int. Neuropsychol. Soc.* 13 246–256 10.1017/S1355617707070270 17286882

[B14] Dziuk M. A. Gidley Larson J. C. Apostu A. Mahone E. M. Denckla M. B. Mostofsky S. H. (2007). Dyspraxia in autism: association with motor, social and communicative deficits. *Dev. Med. Child Neurol.* 49 734–739 10.1111/j.1469-8749.2007.00734.x 17880641

[B15] Fischer M. H. Zwaan R. A. (2008). Embodied language: a review of the role of the motor system in language comprehension. *Q. J. Exp. Psychol.* 61 825–850 10.1080/17470210701623605 18470815

[B16] Fletcher P. T. Whitaker R. T. Tao R. DuBray M. Froehlich A. Ravidchandran C. (2010). Microstructural connectivity of the arcuate fasciculus in adolescents with high-functioning autism. *Neuroimage* 51 1117–1125 10.1016/j.neuroimage.2010.01.083 20132894 PMC2966943

[B17] Fuster J. M. (2003). Cortex and Mind: Unifying Cognition. New York: Oxford University Press

[B18] Gaffrey M. S. Kleinhans N. M. Haist F. Akshoomoff N. Campbell A. Courchesne E. (2007). Atypical participation of visual cortex during word processing in autism: an fMRI study of semantic decision. *Neuropsychologia* 45 1672–1684 10.1016/j.neuropsychologia.2007.01.008 17336346 PMC2071933

[B19] Garaghani M. Wennekers T Pulvermüller F. (2009). A neuroanatomically grounded Hebbian-learning model of attention-language interactions in the human brain. *Eur. J. Neurosci.* 27 492–513 10.1111/j.1460-9568.2008.06015.x PMC2258460 18215243

[B20] Harris G. J. Chabris C. F. Clark J. Urban T. Aharon I. Steele S. (2006). Brain activation during semantic processing in autism spectrum disorders via functional magnetic resonance imaging. *Brain Cogn.* 61 54–68 10.1016/j.bandc.2005.12.015 16473449

[B21] Hauk O. Johnsrude I Pulvermüller F. (2004). Somatotopic representation of action words in human motor and premotor cortex. *Neuron* 41 301–307 10.1016/S0896-6273(03)00838-9 14741110

[B22] Hauk O. Tschentscher N. (2013). The body of evidence: what can neuroscience tell us about embodied semantics? *Front. Psychol.* 4:50 10.3389/fpsyg.2013.00050 PMC3570773 23407791

[B23] Iacoboni M. Dapretto M. (2006). The mirror neuron system and the consequences of its dysfunction. *Nat. Rev. Neurosci.* 7 942–951 10.1038/nrn2024 17115076

[B24] Jansiewicz E. M. Goldberg M. C. Neschaffer C. J. Denckla M. B. Landa R. Mostofsky S. H. (2006). Motor signs distinguish children with high functioning autism and Asperger’s Syndrome from controls. *J. Autism Dev. Disord.* 36 613–621 10.1007/s10803-006-0109-y 16609826

[B25] Jeannerod M. (2006). Motor Cognition: What Actions Tell the Self. New York: Oxford University Press 10.1093/acprof:oso/9780198569657.001.0001

[B26] Jones T. B. Bandettini P. A. Kenworthy L. Case L. K. Milleville S. C. Martin A. (2010). Sources of group differences in functional connectivity: an investigation applied to autism spectrum disorder. *Neuroimage* 49 401–414 10.1016/j.neuroimage.2009.07.051 19646533 PMC2832835

[B27] Kamio Y. Toichi M. (2000). Dual access to semantics in autism: is pictorial access superior to verbal access? *J. Child Psychol. Psychiatry* 41(7) 859–867. 10.1111/1469-7610.00673 11079428

[B28] Kana R. Keller T. A. Cherkassky V. L. Minshew N. J. Just M. A. (2006). Sentence comprehension in autism: thinking in pictures with decreased functional connectivity. *Brain* 129 2484–2493 10.1093/brain/awl164 16835247 PMC4500127

[B29] Keller T. A. Kana R. K. Just M. A. (2007). A developmental study of the structural integrity of white matter in autism. *Neuroreport* 18 23–27 10.1097/01.wnr.0000239965.21685.99 17259855

[B30] Kemmerer D. Rudrauf D. Manzel K. Tranel D. (2012). Behavioural patterns and lesion sites associated with impaired processing of lexical and conceptual knowledge of action. *Cortex* 48 826–848 10.1016/j.cortex.2010.11.001 21159333 PMC3965329

[B31] Kiefer M Pulvermüller F. (2012). Conceptual representations in mind and brain: theoretical developments, current evidence and future directions. *Cortex* 48 805–825 10.1016/j.cortex.2011.04.006 21621764

[B32] Kriegeskorte N. Simmons W. K. Bellgowan P. S. Baker C. I. (2009). Circular analysis in systems neuroscience: the dangers of double dipping. *Nat. Neurosci.* 12 535–540 10.1038/nn.2303 19396166 PMC2841687

[B33] Kronbichler M. Hutzler F. Wimmer H. Mair A. Staffen W. Ladurner G. (2004). The visual word form area and the frequency with which words are encountered: evidence from a parametric fMRI study. *Neuroimage* 21 946–953 10.1016/j.neuroimage.2003.10.021 15006661

[B34] Lai G. Pantazatos S. P. Schneider H. Hirsch J. (2012). Neural systems for speech and song in autism. *Brain* 135 961–975 10.1093/brain/awr335 22298195 PMC3286324

[B35] Leary M. R. Hill D. A. (1996). Moving on: autism and movement disturbance. *Ment. Retard.* 34 39–53 8822025

[B36] Lombardo M. V. Chakrabarti B. Bullmore E. T. Wheelwright S. J. Sadek S. A. Suckling J. (2010a). Shared neural circuits for mentalizing about the self and others. *J. Cogn. Neurosci.* 22 1623–1635 10.1162/jocn.2009.21287 19580380

[B37] Lombardo M. V. Chakrabarti B. Bullmore E. T. Sadek S. k. Pasco G. Wheelwright S. J. (2010b). Atypical neural self-representation in autism. *Brain* 133 611–624 10.1093/brain/awp306 20008375

[B38] Lombardo M. V. Baron-Cohen S. (2010). Unraveling the paradox of the autistic self. *WIREs Cogn. Sci.* 1 393–403 10.1002/wcs.45 26271379

[B39] Lombardo M. V. Baron-Cohen S. (2011). The role of the self in mindblindness in autism. *Conscious. Cogn.* 20 130–140 10.1016/j.concog.2010.09.006 20932779

[B40] Mahon B. Z. Caramazza A. (2008). A critical look at the embodied cognition hypothesis and a new proposal for grounding conceptual content. *J. Physiol. Paris* 102(1–3) 59–70 10.1016/j.jphysparis.2008.03.004 18448316

[B41] Marsh L. E Hamilton A. F. C. (2011). Dissociation of mirroring and mentalising systems in autism. *Neuroimage* 56 1511–1519 10.1016/j.neuroimage.2011.02.003 21310248

[B42] Mostofsky M. P. Burgess M. P Gidley Larson J. C. (2006). Increased motor cortex white matter volume predicts motor impairment in autism. *Brain* 8 2117–2122 10.1093/brain/awm129 17575280

[B43] Moseley R. Carota F. Hauk O. Mohr B Pulvermüller F. (2012). A role for the motor system in binding abstract emotional meaning. *Cereb. Cortex* 22 1634–1647 10.1093/cercor/bhr238 21914634 PMC3377965

[B44] Moseley R. L. Pulvermüller F. Mohr B. Lombardo M. V. Baron-Cohen S. Shtyrov Y. (2013). Brain routes for reading in adults with and without autism: EMEG evidence. *J. Autism Dev. Disord.*. 10.1007/s10803-013-1858-z [Epub ahead of print]. PMC3898534 23748435

[B45] Mostofsky M. P. Ewen J. (2011). Altered connectivity and action model formation in autism is autism. *Neuroscientist* 17 437–448 10.1177/1073858410392381 21467306 PMC3974163

[B46] Mottron L. Dawson M. Soulieres I. Hubert B. Burack J. (2006). Enhanced perceptual functioning in autism: an update, and eight principles of autistic perception. *J. Autism Dev. Disord.* 36 27–43 10.1007/s10803-005-0040-7 16453071

[B47] Neininger B Pulvermüller F. (2003). Word-category specific deficits after lesions in the right hemisphere. *Neuropsychologia* 41 53–70 10.1016/S0028-3932(02)00126-4 12427565

[B48] Piaget J. (1950). The Psychology of Intelligence. London: Routledge

[B49] Pulvermüller F. (1999). Words in the brain’s language. *Behav. Brain Sci.* 22 253–336 10.1017/S0140525X9900182X 11301524

[B50] Pulvermüller F. (2005). Brain mechanisms linking language and action. *Nat. Rev. Neurosci.* 6 576–582 10.1038/nrn1706 15959465

[B51] Pulvermüller F. (2013). How neurons make meaning: brain mechanisms for embodied and abstract-symbolic semantics. *Trends Cogn. Sci. (Regul. Ed.)* 17 458–470 10.1016/j.tics.2013.06.004 23932069

[B52] Pulvermüller F. Fadiga L. (2010). Active perception: sensorimotor circuits as a cortical basis for language. *Nat. Rev. Neurosci.* 11 351–360 10.1038/nrn2811 20383203

[B53] Pulvermüller F. Hauk O. Nikulin V. V. Ilmoniemi R. J. (2005). Functional links between motor and language systems. *Eur. J. Neurosci.* 21 793–797 10.1111/j.1460-9568.2005.03900.x 15733097

[B54] Rizzolatti G. Craighero L. (2004). The mirror neuron system. *Annu. Rev. Neurosci.* 27 169–192 10.1146/annurev.neuro.27.070203.144230 15217330

[B55] Rizzolatti G. Fabbri-Destro M. (2010). Mirror neurons: from discovery to autism. *Exp. Brain Res.* 200 223–237 10.1007/s00221-009-2002-3 19760408

[B56] Rogers S. J. (2009). What are infant siblings teaching us about autism in infancy? *Autism Res.* 2 125–137 10.1002/aur.81 19582867 PMC2791538

[B57] Schippers M. B. Roebroeck A. Renken R. Nanetti L. Keysers C. (2010). Mapping the information flow from one brain to another during gestural communication. *Proc. Natl. Acad. Sci. U.S.A.* 107 9388–9393 10.1073/pnas.1001791107 20439736 PMC2889063

[B58] Shebani Z Pulvermüller F. (2013). Moving the hands and feet specifically impairs working memory for arm- and leg-related action words. *Cortex* 49 222–231 10.1016/j.cortex.2011.10.005. 22113187

[B59] Spunt R. P. Lieberman M. D. (2012). Dissociating modality-specific and supramodal neural systems for action understanding. *J. Neurosci.* 32 1–9 10.1523/JNEUROSCI.5715-11.2012 22399779 PMC6621054

[B60] Sundaram S. K. Kumar A. Makki M. M. Behen M. E. Chugani H. T. Chugani D. C. (2008). Diffusion tensor imaging of frontal lobe in autism spectrum disorder. *Cereb. Cortex* 18 2659–2665 10.1093/cercor/bhn031 18359780 PMC2567426

[B61] Teitelbaum P. Teitelbaum O. Nye J. Fryman J. Maurer R. G. (1998). Movement analysis in infancy may be useful for early diagnosis of autism. *Proc. Natl. Acad. Sci. U.S.A.* 95 13982–13987 10.1073/pnas.95.23.13982 9811912 PMC25000

[B62] Toichi M. Kamio Y. (2001). Verbal association for simple common words in high-functioning autism. *J. Autism Dev. Disord.* 31 483–490 10.1023/A:1012216925216 11794413

[B63] Toichi M. Kamio Y. (2002). Long-term memory and levels-of-processing in autism. *Neuropsychologia* 40 964–969 10.1016/S0028-3932(01)00163-4 11900748

[B64] Toichi M. Kamio Y. (2003). Long-term memory in high-functioning autism: controversy on episodic memory in autism reconsidered. *J. Autism Dev. Disord.* 33 151–161 10.1023/A:1022935325843 12757354

[B65] Vigliocco G. Vinson D. P. Druks J. Barber H. Cappa S. F. (2011). Nouns and verbs in the brain: a review of behavioural, electrophysiological, neuropsychological and imaging studies. *Neurosci. Biobehav. Rev.* 35 407–426 10.1016/j.neubiorev.2010.04.007 20451552

[B66] Williams J. H. G. (2008). Self-other relations in social development and autism: multiple roles for mirror neurons and other brain bases. *Autism Res.* 1 73–90 10.1002/aur.15 19360654

[B67] Williams J. H. G. Whiten A. Suddendorf T. Perrett D. I. (2001). Imitation, mirror neurons and autism. *Neurosci. Biobehav. Rev.* 25 287–295 10.1016/S0149-7634(01)00014-8 11445135

[B68] Wilson S. M. Saygin A. P. Sereno M. I. Iacoboni M. (2004). Listening to speech activates motor areas involved in speech production. *Nat. Neurosci.* 7 701–702 10.1038/nn1263 15184903

[B69] Woodbury-Smith M. R. Robinson J. Wheelwright S. Baron-Cohen S. (2005). Screening adults for Asperger syndrome using the AQ: a preliminary study of its diagnostic validity in clinical practice. *J. Autism Dev. Disord.* 35 331–335 10.1007/s10803-005-3300-7 16119474

[B70] Zaki J. Weber J. Bolger N. Oschner K. (2009). The neural bases of empathic accuracy. *Proc. Natl. Acad. Sci. U.S.A.* 106 11382–11387 10.1073/pnas.0902666106 19549849 PMC2708723

